# A novel polymer platform for endoscopic tattooing with high efficacy and safety

**DOI:** 10.3389/fbioe.2024.1409681

**Published:** 2024-07-05

**Authors:** Liang Zhang, Mengni Jiang, Zheng Chen, Xinyuan Zhang, Wei An, Shige Wang, Jiulong Zhao

**Affiliations:** ^1^ Department of Gastroenterology, Changhai Hospital, Naval Medical University, Shanghai, China; ^2^ School of Materials and Chemistry, University of Shanghai for Science and Technology, Shanghai, China

**Keywords:** endoscopy, endoscopic tattooing, colonoscopy, tattoo dye, polypyrrole

## Abstract

Endoscopic tattooing plays a pivotal role in modern endoscopic localization of gastrointestinal lesions, facilitating further surgical intervention and aiding in the postoperative identification and repositioning of lesions. However, traditional endoscopic tattoo dyes often suffer from drawbacks such as side effects, short tattoo duration, and high overall costs. In this study, we developed polyvinylpyrrolidone (PVP)-modified polypyrrole (PPy) nanoparticles by oxidizing pyrrole in a PVP aqueous solution to create a PPy/PVP nanoparticle solution. This innovation aims to enhance endoscopic tattooing efficiency and mitigate the limitations associated with current tattooing methods. Both *in vitro* and *in vivo* evaluations confirmed the biosafety of PPy/PVP nanoparticles. Endoscopic tattooing experiments conducted in a pig model demonstrated the dye’s stability within the digestive tract. Similarly, subcutaneous tissue tattooing experiments performed in a mouse model revealed the sustained stability of the PPy/PVP tattoo dye for at least 180 days. With its robust stability, safety, and longevity, PPy/PVP nanoparticles hold promise as novel tattoo dyes for marking intestinal lesion sites. This advancement has the potential to enhance the accuracy of lesion localization and long-term tracking.

## Introduction

Endoscopic tattooing, also known as endoscopic marking or endoscopic tattoo placement, is a medical procedure used to mark specific locations within the gastrointestinal (GI) tract and, more recently, even for marking pancreatic lesions to be referenced during endoscopic procedures or surgeries (Beretvas and Ponsky, 2001; Luigiano et al., 2012). The purpose of this technique (Kethu et al., 2010) is to provide a visible and permanent marker within the GI tract, aiding in the navigation and identification of specific sites during subsequent medical interventions (Yang et al., 2017; Barberio et al., 2020). During the endoscopic tattooing procedure, a small amount of sterile ink or dye is injected into the tissue lining of the GI wall using an endoscope (Snider et al., 1994; Ashida et al., 2006; Barberio et al., 2020). The tattooing helps in marking areas of interest, such as polyps, tumors, regions of inflammation, or other abnormalities (Kluger et al., 2008). Endoscopic tattooing is commonly employed in cases where precise localization is crucial, such as when planning for the surgical removal of a specific lesion or monitoring the progression of a particular condition over time (Botoman et al., 1994). This technique enhances the accuracy and efficiency of medical interventions in the GI system (Beretvas and Ponsky, 2001; Barberio et al., 2020). The marking created by tattooing helps guide subsequent procedures, such as surgical resection or follow-up endoscopies, by providing a visible reference point within the GI tract (Snider et al., 1994).

There are several types of dyes or inks used for endoscopic tattooing. India ink is a traditional choice for endoscopic tattooing. It is a dark, carbon-based ink that provides a long-lasting and visible marker within the GI tract. India ink is often preferred for its permanence and ease of visualization during subsequent procedures (Gianom et al., 2003; Dell'Abate et al., 1999). Carbon particle suspensions are often used as an alternative to India ink. Sterile Carbon Particle Suspension is a dye consists of sterile carbon particles suspended in a solution. It is similar to India ink in terms of its dark color and long-lasting effect. Researchers have enhanced the components found in India Ink and developed a substance named Spot^®^ (GI Supply, Camp Hill, PA, United States), comprising exceptionally purified carbon particles for tattooing purposes. SPOT^®^, the only drug approved by the FDA for Endoscopic tattooing and is considered safety and durable, aims to mitigate the slight tissue inflammation associated with conventional India Ink (Askin et al., 2002). However, its high cost has hindered its widespread clinical use (Slof and Gras, 2012; Rizi et al., 2015). Synthetic dyes, such as methylene blue or indigo carmine, may also be used for endoscopic tattooing (Cambruzzi et al., 1985; Beretvas and Ponsky, 2001). Indocyanine green, another marker, is famous for its excellent staining and durability in endoscopic tattooing of colon, with no adverse tissue reactions (Hammond et al., 1989; Miyoshi et al., 2009). These dyes offer the advantage of being water-soluble, which can aid in their distribution and visibility within the GI tract. However, synthetic dyes may not be as permanent as carbon-based inks (Milone et al., 2019). In some cases, the tattoo dye may migrate from its original injection site within the GI tract (Snider et al., 1994; Cappell et al., 2010). This migration can make it challenging to accurately locate the marked area during subsequent procedures, potentially leading to complications or incomplete treatment (Imai et al., 2016; Rodrigues et al., 2018).

While the permanence of the tattoo dye is advantageous for long-term follow-up, it can also pose challenges if the marked area needs to be removed or future endoscopy. Removing tattoo dye from the GI tract can be technically challenging (Park et al., 1991; Singh et al., 2006). Depending on the type of dye used and the characteristics of the GI tissue, the visibility of the tattoo mark may vary. Moreover, factors such as tissue inflammation, scarring, or the presence of blood can affect the clarity and visibility of the tattoo during subsequent procedures (Price et al., 2000). Considering these potential drawbacks of current endoscopic tattooing solutions, designing a new type of tattooing dye that is biologically safe and long-lasting visible under endoscopy is scientific sounding and has important clinical values.

For this purpose, we designed an endoscopic tattooing dye based on polyvinylpyrrolidone (PVP) coated polypyrrole (PPy) nanoparticules. PPy, formed through oxidative self-polymerization of pyrrole in the PVP solution, was synchronously modified with PVP. Our results showed that utilizing such PPy/PVP as an endoscopic tattooing dye not only ensures a long-lasting localization effect but also ensures high biosafety, causing minimal *in vivo* adverse reactions. Additionally, PPy is cost-effective and can be produced on a large scale, which may facilitate its potential clinical translation. As a result, as a novel candidate for endoscopic tattooing, PVP/PPy provides a hopeful outlet for designing dyes with cost-effectiveness, biosafety, and prolonged durability.

## Methods

### Materials

Pyrrole (99%) was procured from Titan Technology Co., Ltd. (Shanghai, China). PVP [(C_6_H_9_NO)n, V900010, MW = 360,000D] was sourced from Sigma‒Aldrich (Shanghai, China). Ferric chloride hexahydrate and sodium hydroxide (AR, 96%) were obtained from Aladdin Bio-Chem Technology Co., Ltd. (Shanghai, China). The Cell Counting Kit-8 (CCK-8) (CK04) and Live/Dead Cell Staining Kit (C2015M) were acquired from Beyotime Biotechnology (Shanghai, China) Co. Ltd. For endoscopic procedures, the disposable injection needle (NM-200U-0423) was supplied by Olympus (Japan), while the esophagoscope and gastroscope utilized were Olympus GIF-HQ290 models, and the enteroscope employed was an Olympus CF-HQ290L/I variant.

### Synthesis and characterizations of PPy/PVP

Take 0.5 g of PVP dissolved in 100 mL of deionized water and stirred well at room temperature to obtain PVP aqueous solution. Then, 40 mL of PVP aqueous solution was taken in a glass vial, which was placed in an ice water bath for cooling. 2.48 g of ferric chloride hexahydrate and 140 μL of pyrrole (Shanghai Titan Technology Co., Ltd., concentration: 99%) were added sequentially into the glass vial, and the conjugated polymer matrix composite polypyrrole (PPy) for tattooing was obtained after 4 h of reaction. Upon completion of the reaction, the resulting PPy/PVP, serving as an effective tattooer, was produced and stored at 37°C for future use.

### Characterization

The hydrodynamic diameters were measured using a ZetaSizer Nano-ZS 90 instrument (Malvern Instruments, Malvern, United Kingdom). X-ray diffraction (XRD) analysis was performed using a Bruker D2 Phaser instrument (Germany). PPy-PVP NPs were observed by the scanning electron microscopy (SEM, FEI Magellan 400).

### Cell culture

Mouse fibroblasts (L929) were procured from the National Collection of Authenticated Cell Cultures (Shanghai, China) and cultured under standard conditions at 37°C with a 5% carbon dioxide (CO_2_) atmosphere. The L929 cells used in experiments were immortalized cell lines, and to ensure the experimental results, only used cells from the first 10 generations. The culture medium employed was Dulbecco’s Modified Eagle Medium (DMEM, Gibco, CA, United States), which was supplemented with 10% (v/v) Fetal Bovine Serum (FBS, Gibco, CA, United States) and 1% penicillin and streptomycin (MedChemExpress, Princeton, United States).

### Cytotoxicity assay

L929 cells were distributed into 96-well plates at a density of 1 × 10^4^ cells per well. Following an overnight incubation, the culture medium was replaced with varying concentrations of PPy/PVP (0 μg/mL, 15 μg/mL, 30 μg/mL, 60 μg/mL, 125 μg/mL, and 250 μg/mL) in complete DMEM, and cells were cultured for an additional 24 h. After the designated incubation period, a solution of 1% calcein-AM and PI dye (100 μL per well) was introduced for live/dead cell staining. Following a 15-min incubation at 37°C, cellular fluorescence was observed using a fluorescence inverted microscope (Leica DM IL LED inverted laboratory microscope, Leica, Germany). Cell viability was evaluated using the CCK-8 assay, wherein 10 μL of CCK-8 solution was added to each well, resulting in a final concentration of 10%. The plates were then incubated at 37°C for 2 h. Absorbance readings at 450 nm were obtained using a microplate reader (Molecular Devices SpectraMax^®^ i3, United States). Each experimental condition was represented by three replicate wells, and cell viability was determined using the following formula:

$$\text{Cell viability}=\frac{{\text{OD}}_{\text{value}\;\text{of}\;\text{experimental}\;\text{group}}-{\text{OD}}_{\text{value}\;\text{of}\;\text{blank}\;\text{group}}}{{\text{OD}}_{\text{value}\;\text{of}\;\text{control}\;\text{group}}-{\text{OD}}_{\text{value}\;\text{of}\;\text{blank}\;\text{group}}}\times 100\%$$

### Hemolysis evaluation

Fresh blood obtained from mice was subjected to centrifugation at 5,000 rpm for 5 min to eliminate the supernatant. Following this, the collected 2 mL of blood was diluted to 50 mL with saline and preserved at 4°C. For experimental purposes, 300 μL of the diluted blood cells were mixed with 1.2 mL of saline and deionized water separately, serving as negative and positive controls, respectively. Additionally, 300 μL of blood cells diluted were combined with 1.2 mL of PPy/PVP at different concentrations (0, 500, 1,000, 1,500, and 2000 ppm in saline). The mixture obtained was subsequently placed in an incubator set at 37°C for a duration of 2 h.

The absorbance of the supernatant was assessed at 540 nm using a microplate reader (Molecular Devices SpectraMax^®^ i3, United States). Subsequently, the hemolysis rate was determined using the following formula:

$$\text{Hemolysis rate }\left(\%\right)=\frac{\;\left({\text{OD}}_{\text{experimental}\;\text{group}}\;-\;{\text{OD}}_{\text{negative}\;\text{control}\;\text{group}}\right)}{\left({\text{OD}}_{\text{positive}\;\text{control}\;\text{group}}\;-\;{\text{OD}}_{\text{negative}\;\text{control}\;\text{group}}\right)}\times 100\%$$

### Animal studies

We obtained three male KM mice (6–8 weeks old) from JieSiJie Laboratory Animal Co., Ltd. (Shanghai, China). Each mouse was subjected to skin tattooing with PPy/PVP under anesthesia. Three Bama pigs (male, 25–30 kg) were procured from Laiboorganism Biotechnology Co., Ltd. (Jiangxi, China) and housed at the Animal Experimental Center of the Naval Medical University. The sample size was selected to uphold the principles of animal ethics regulation, specifically emphasizing replacement, reduction, and refinement, thereby ensuring the reproducibility of the experiments. Each pig underwent endoscopic tattooing with PPy/PVP under anesthesia, targeting the esophagus, stomach, and rectum, respectively. Anesthesia induction involved intramuscular administration of ketamine (25 mg/kg), atropine (0.04 mg/kg), and azaperone (4 mg/kg). Subsequently, isoproterenol (1.66 mg/kg) was administered for anesthesia maintenance, with 1%–2.5% isoflurane inhalation.

Following the administration of general anesthesia, the pigs were positioned on their sides, and utilizing endoscopic visualization as a guide, a needle was inserted into the mid-esophagus, the greater curvature of the stomach, and the rectum (3 cm from the anus) of each of the three pigs using an endoscopic catheter at a 45° angle. The PPy/PVP solution (4 mg/mL, 0.1 mL) was then injected using a syringe needle pre-filled with the solution. Endoscopic examinations were conducted on days 3, 7, and 24 post-tattooing to monitor the status of the tattooing. Following the final endoscopy, the pigs were euthanized, and markers from the tattoo sites in the esophagus, stomach, and rectum were collected and subjected to H&E staining. Skin samples were collected from the corresponding sites on the backs of 3 KM mice 180 days after dorsal skin tattoos and stained for H&E and Masson to evaluate pathologic changes. Additionally, heart, liver, spleen, lungs, and kidneys were collected from both pigs and KM mice for H&E staining to assess the safety of PPy/PVP in animals. All staining procedures were performed following standard protocols.

### Statistical analysis

All values are reported as mean ± SD with the indicated sample size. *p < 0.05, **p < 0.01, ***p < 0.001, and ****p < 0.0001 represented different statistical significances. The data statistical analysis was performed using GraphPad Prism 9.5. The ns stands for no significant difference.

### Ethics approval

All the animal experiments were performed with the approval of the Naval Medical University Experimental Animal Center and all procedures were performed according to the guidelines and animal welfare protocols.

## Results and discussions

### Characterization of PPy/PVP

**FIGURE 1:**
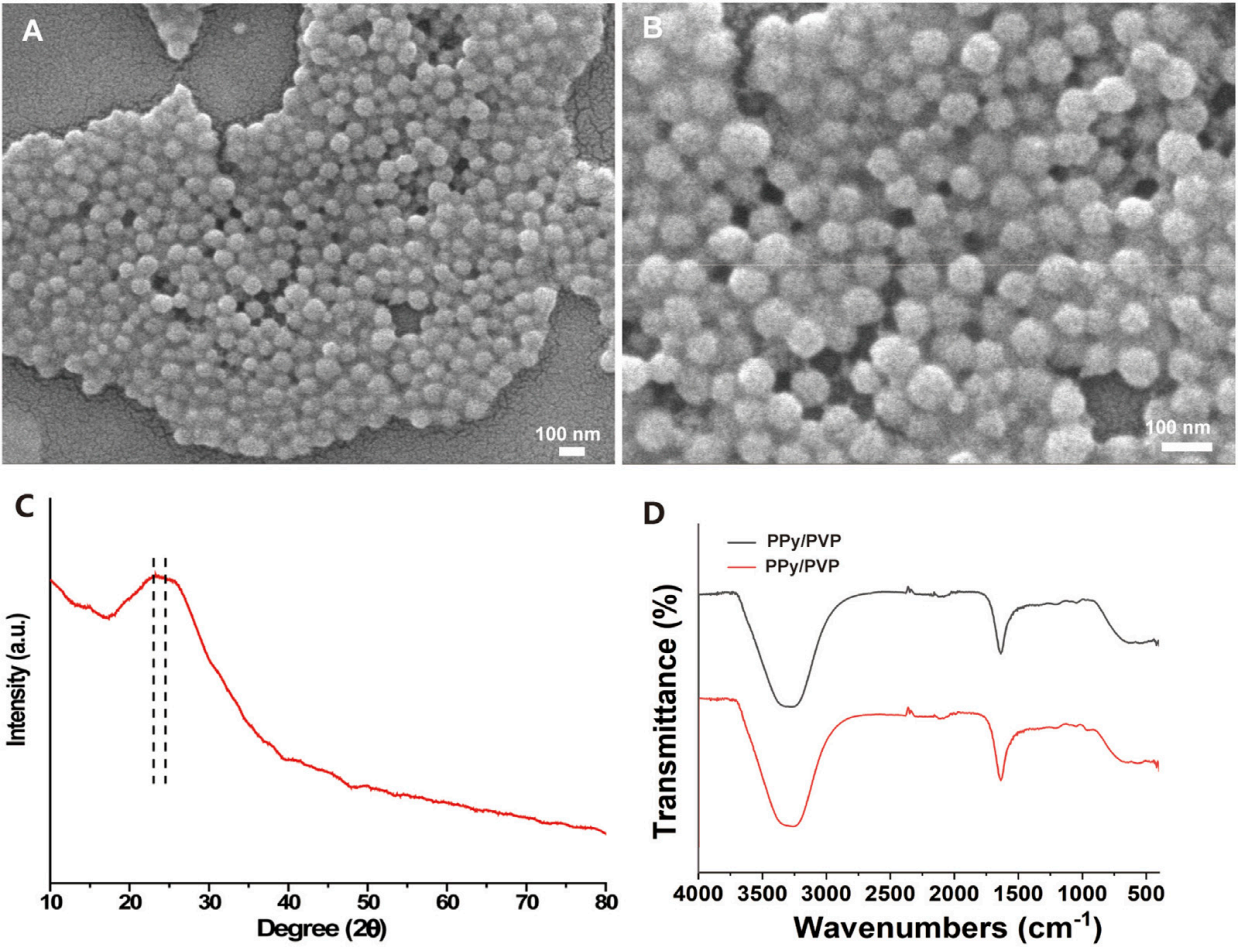
**(A and B)** SEM image of PPy/PVP nanoparticle. **(C)** XRD image of PPy/PVP nanoparticle. **(D)** FTIR image of PPy/PVP nanoparticle.

In this study, we developed a PVP-modified PPy nanoparticle (PPy/PVP) as a dye for endoscopic tattooing. As observed by SEM, the PPy/PVP Nanoparticles were homogeneous in morphology with an average particle size of 81.78 ± 6.82 nm and good monodispersity (Figures 1A,B). The spherical shape of PPy/PVP nanoparticles and the absence of typical diffraction features in the XRD spectra of PPy (Figure 1C) confirmed its amorphous structure. The FTIR analysis of PPy/PVP reveals successful modification of PVP molecules onto the surface of PPy. Peaks located at 1,542 cm^−1^, 1,230 cm^−1^ and 1,051 cm^−1^ were detected from the spectra of PPy-PVP NPs, which correspond to the characteristic C-C conjugation, C-H in-plane bending and C-H ring deformation of PPy, respectively. The vibrational signals at 1,637 cm^−1^, 1,488 cm^−1^, and 1,303 cm^−1^ can be attributed to the CO-stretching vibration, C-N-stretching vibration, and -CH_2_ deformation vibration of the PVP, respectively, which clearly indicate that the PVP has been successfully adhered to the surface of PPy NPs. The peak at 3,264 cm^−1^ can be attributed to the characteristic peak of bound water (Figure 1D). The incorporation of PVP enhanced the biocompatibility and colloidal stability of PPy under physiological conditions.

**FIGURE 2:**
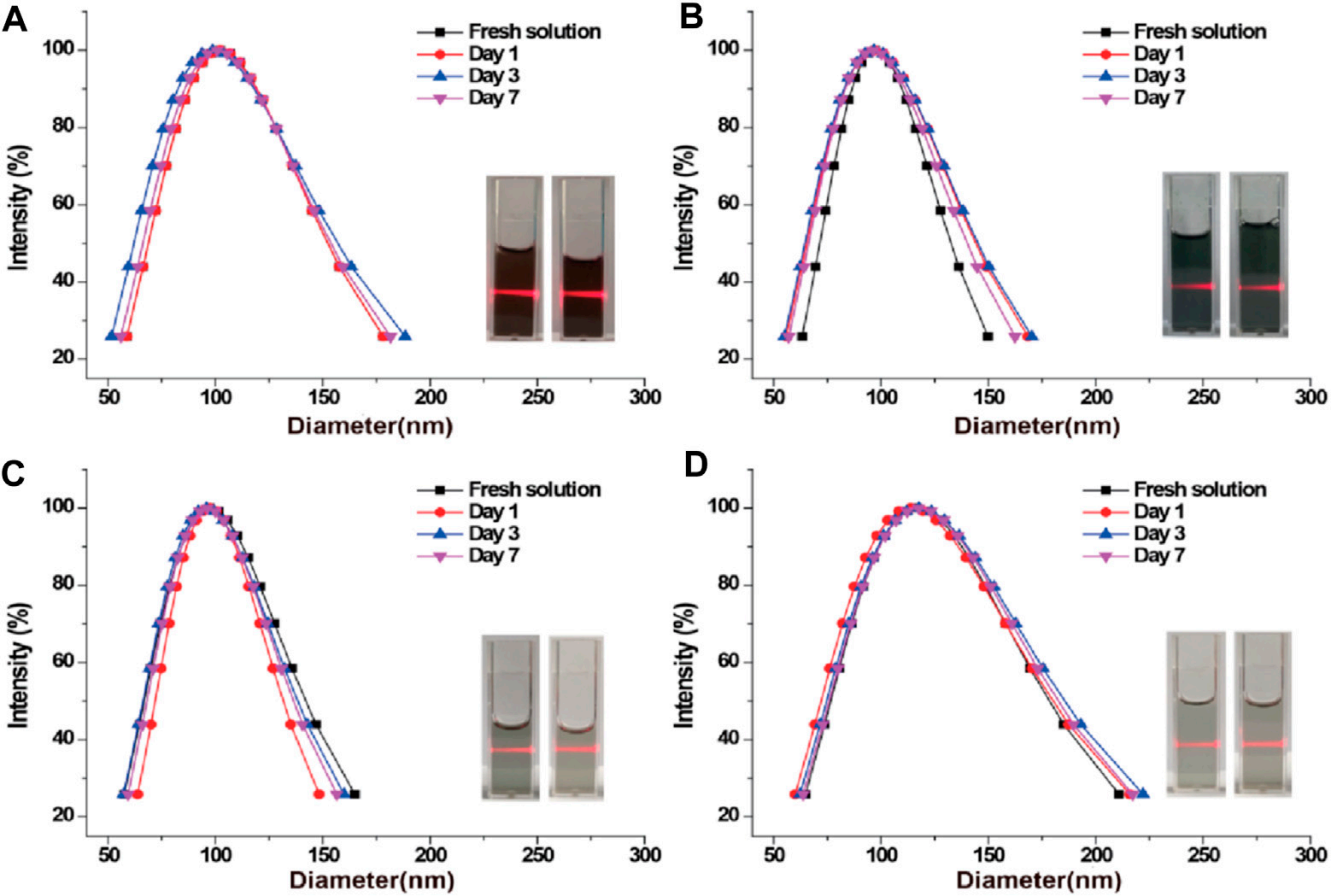
DLS and photographic images of PPy/PVP nanoparticles in **(A)** DMEM, **(B)** PBS, **(C)** saline, and **(D)** DI water, respectively.

Further studies on the stability of PPy/PVP included evaluating its hydrodynamic diameter in different solutions at different time points. The results showed that PPy/PVP exhibited strong stability in different solutions including DMEM, PBS, saline and deionized water (Figures 2A–D). In addition, PPy/PVP exhibited a significant Tyndall effect in these media. This observation further proves the colloidal stability of PPy/PVP in different solutions. The above results demonstrated the successful synthesis of stable PPy/PVP. This black solution showed long-term colloidal stability in DMEM, PBS, saline and DI water and can be used as a stable nanostain of choice for endoscopic tattooing.

### *In vitro* biocompatibility and hemocompatibility

**FIGURE 3:**
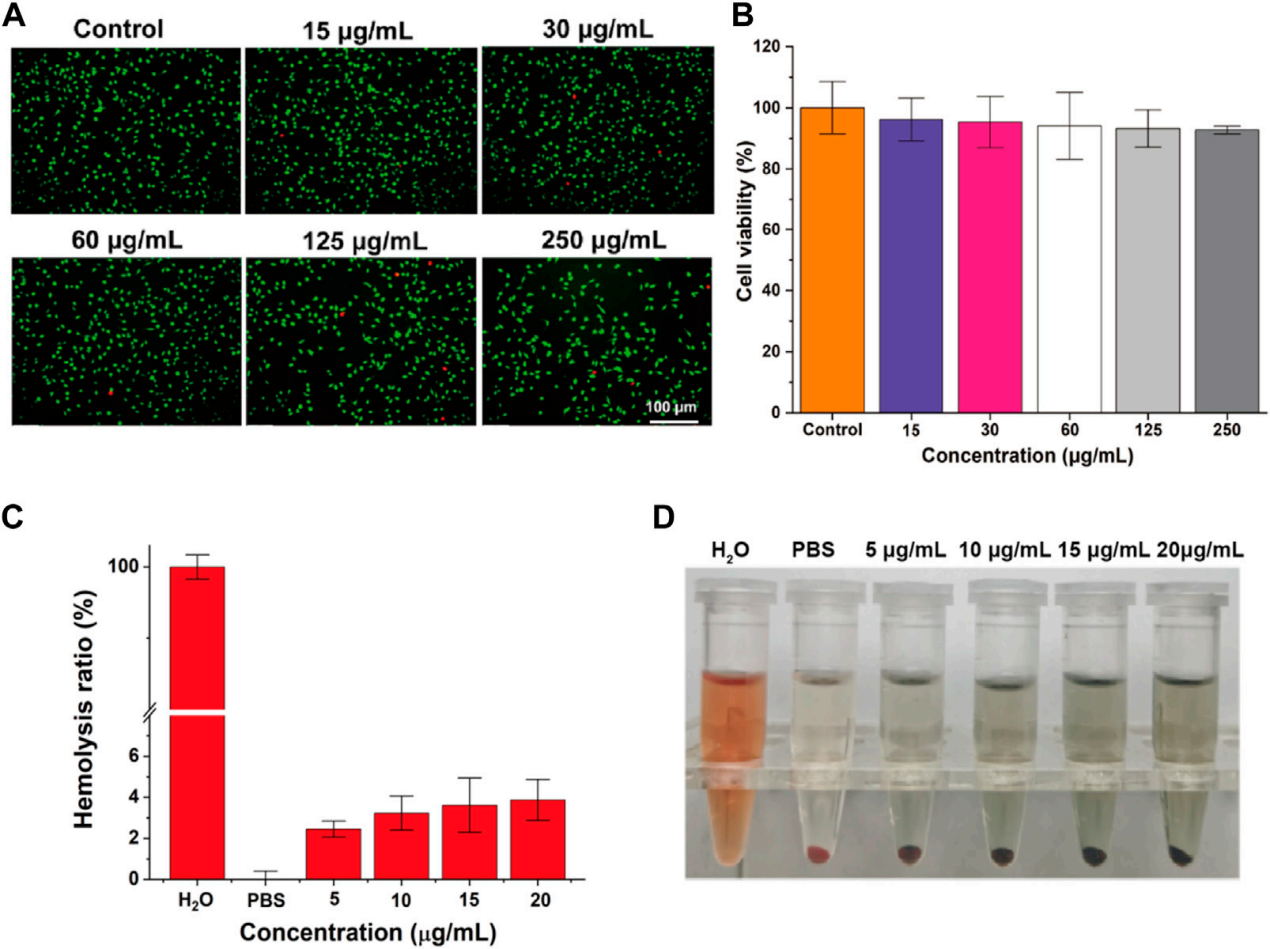
**(A)** Live/dead cell staining of L929 cells after cocultured with different concentrations of PPy/PVP. **(B)** The viability of L929 cells after co-culture of PPy/PVP at different concentrations. **(C)** Hemolysis rate of water, PBS, and PPy/PVP at concentrations of 5, 10, 15, and 20 μg/mL. Data are presented as the mean ± SD (*n* = 3). **(D)** From left to right: picture of water, PBS, and PPy/PVP (at concentrations of 5, 10, 15, and 20 μg/mL)-treated blood cells.

The *in vitro* biocompatibility serves as a prerequisite for further investigating the tattooing effectiveness of the material at the *in vivo* level. The viability of L929 cells was evaluated through live/dead cell staining and CCK-8 analysis following 24 h of co-incubation with varying concentrations of PPy/PVP. Microscopic inspection (Figure 3A) revealed predominantly green fluorescence in L929 cells when viewed under an inverted fluorescence microscope, even after exposure to different concentrations of PPy/PVP (0, 15, 30, 60, 125, and 250 μg/mL). Moreover, findings from the CCK-8 assay (Figure 3B) consistently indicated that the viability of L929 cells remained above 90%, even when exposed to a high concentration of 250 μg/mL of PPy/PVP. This observation underscores the cellular safety profile of PPy/PVP. Further examination of hemocompatibility (Figure 3C) demonstrated that the hemolysis rate remained consistently below 5% at PPy/PVP concentrations ranging from 5 to 20 μg/mL, thus confirming the favorable hemocompatibility of PPy/PVP. Blood erythrocytes treated with water showed almost complete destruction, suggesting no significant non-specific reaction between the erythrocytes and PPy/PVP nanoparticles. Additionally, the absence of notable hemolysis in the experimental group, as indicated in the inset, demonstrates the excellent *in vitro* hemocompatibility of PPy-PVP nanoparticles within the tested concentration range (Figure 3D).

### *In vivo* biocompatibility and tattoo duration in pig model

**SCHEME 1:**
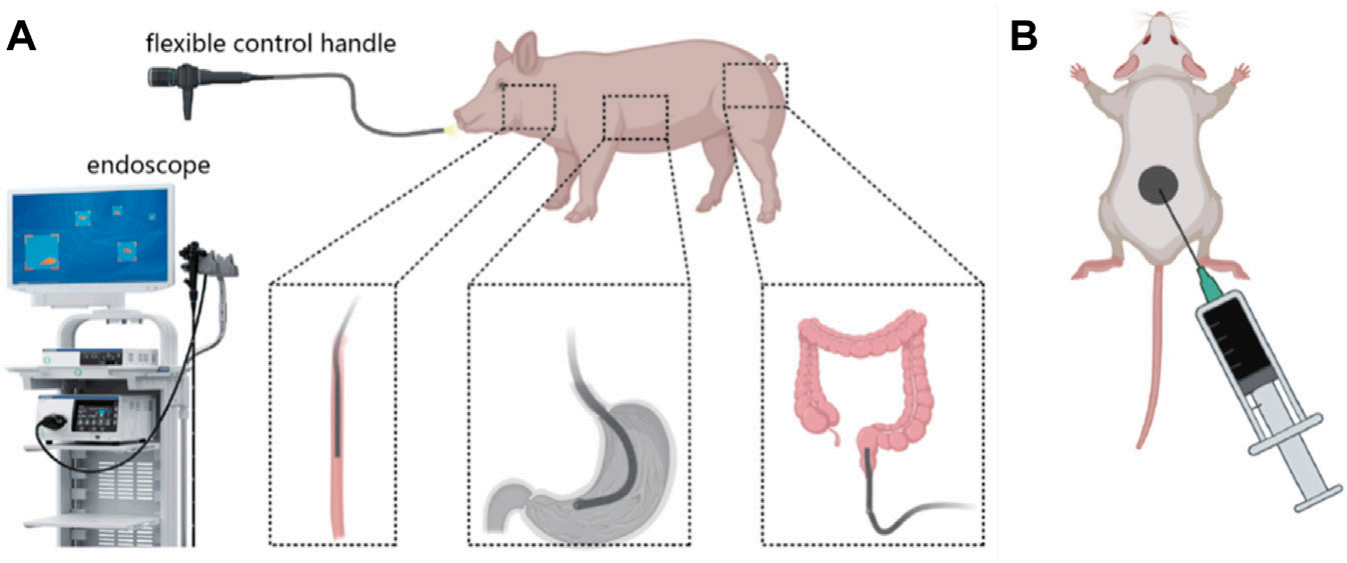
**(A)** Schematic diagram of using PPy/PVP to tattoo the esophagus, stomach, and rectum of a pig under endoscopy. **(B)** Schematic diagram of using PPy/PVP to tattoo the skin tissue of KM mice.

To comprehensively assess the biocompatibility and durability of PPy/PVP as an endoscopic tattooing agent, we conducted evaluations in pig models, aiming to provide thorough insights into its practical efficacy and safety profile. Endoscopic tattooing was performed in key anatomical sites including the esophagus, gastric body, and rectum (3 cm from the anus) of pigs (Scheme 1A). Further, we administered an equivalent dosage of PPy/PVP into the skin tissue of KM mice for 180 days of post-tattooing (Scheme 1B).

**FIGURE 4:**
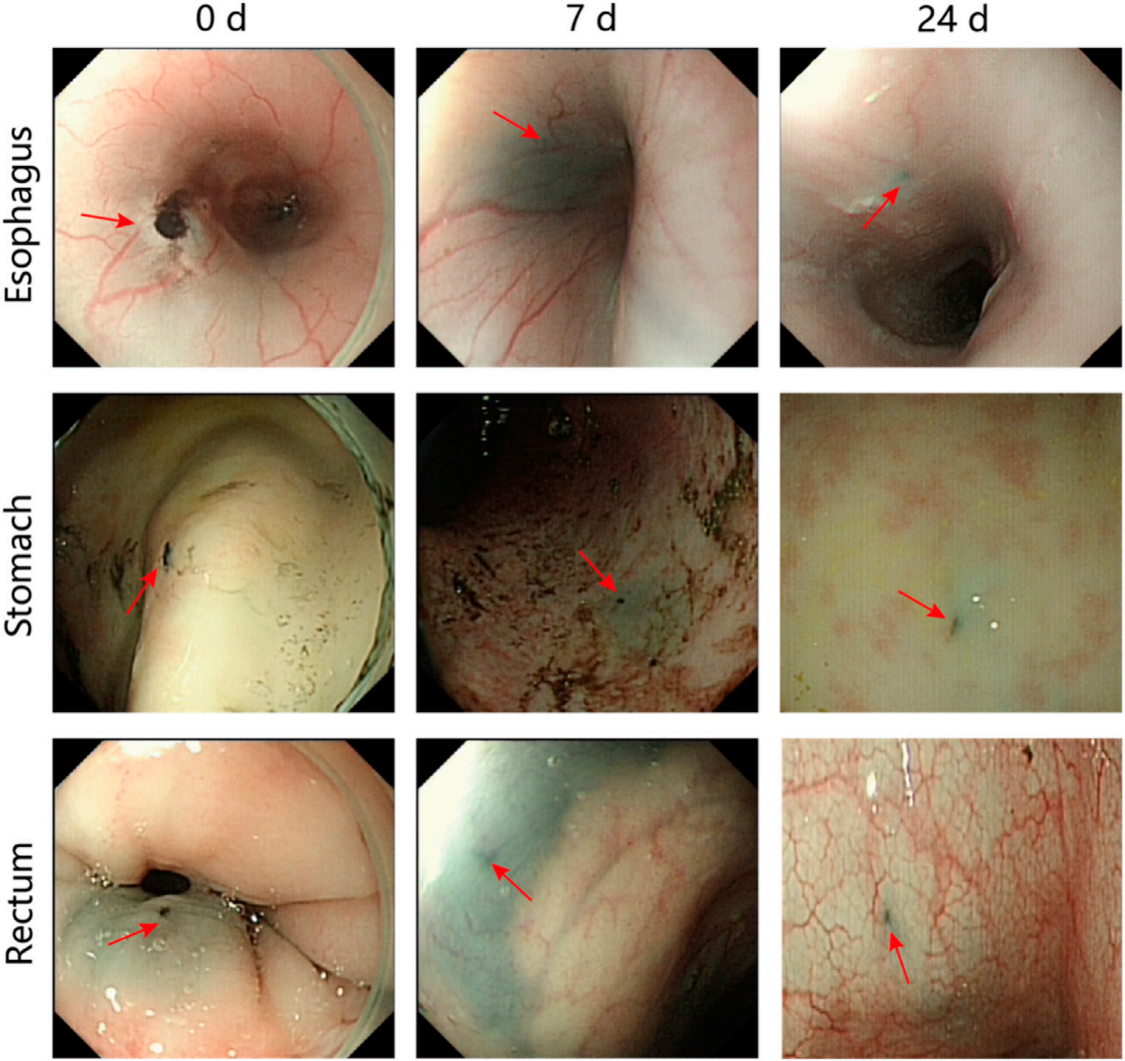
Endoscopic images after days 0, 7, and 24 following the use of PPy/PVP tattoos (red arrow) in the esophagus, stomach, and rectum in a pig model.

We evaluated the effect of PPy/PVP on tattooing by endoscopic observation of the tattooed area of the pig. Subsequently, we performed endoscopic evaluations on days 3, 7, and 24 after tattooing. The results of submucosal injection of PPy/PVP showed obvious black staining at the injection site (shown by the red arrow in Figure 4). Even 7 days after the tattoo, the black spots were still evident. The black material persisted in the submucosal layers of the esophagus, stomach, and rectum, albeit with a slight discoloration at day 24.

**FIGURE 5:**
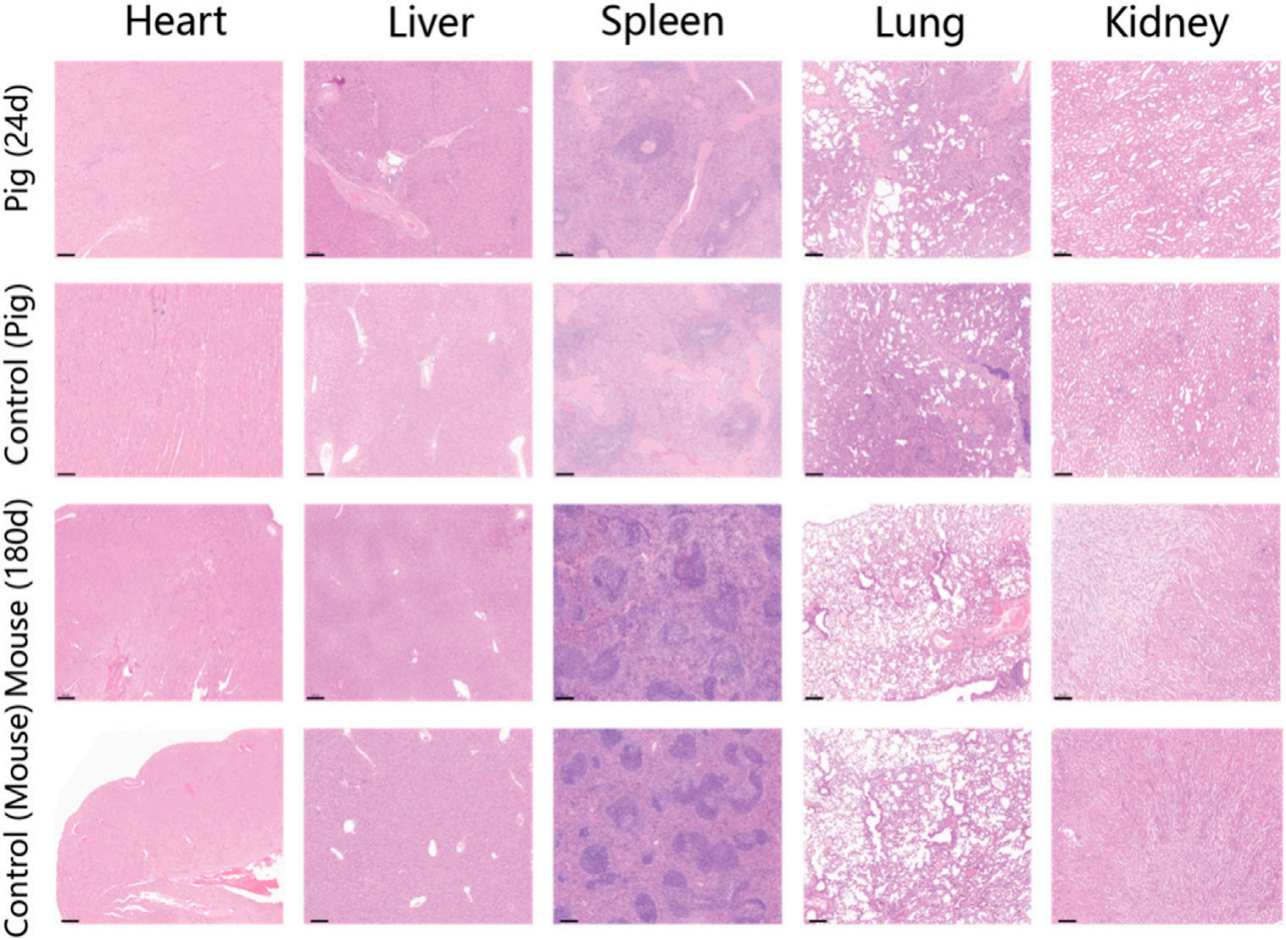
H&E staining results of the heart, liver, spleen, lung, and kidney of the pig after endoscopic tattooing for 24 days. The second line shows the H&E staining results of the heart, liver, spleen, lung, and kidney of a pig without endoscopic tattooing. The third line shows the results of heart, liver, spleen, lung, and kidney of KM mice after skin tissue tattooing for 180 days. The last line shows the H&E staining results of the heart, liver, spleen, lung, and kidney of mice without skin tissue tattooing. Bars represent 200 µm.

At the conclusion of the 24-day endoscopic follow-up, the pigs were humanely euthanized, and H&E staining of the tattooed sites was conducted (Figure 5). The findings revealed minimal inflammatory cell infiltration near the tattooed sites, indicating the favorable endobiological compatibility of PPy/PVP during tattooing. Furthermore, compared to pigs and mice without endoscopic tattooing or skin tissue tattooing, the H&E staining results of major organs such as the heart, liver, spleen, lungs, and kidneys showed no significant damage after 24 days in pigs and 180 days in KM mice (Figure 5), thus confirming the excellent *in vivo* biocompatibility of PPy/PVP.

**FIGURE 6:**
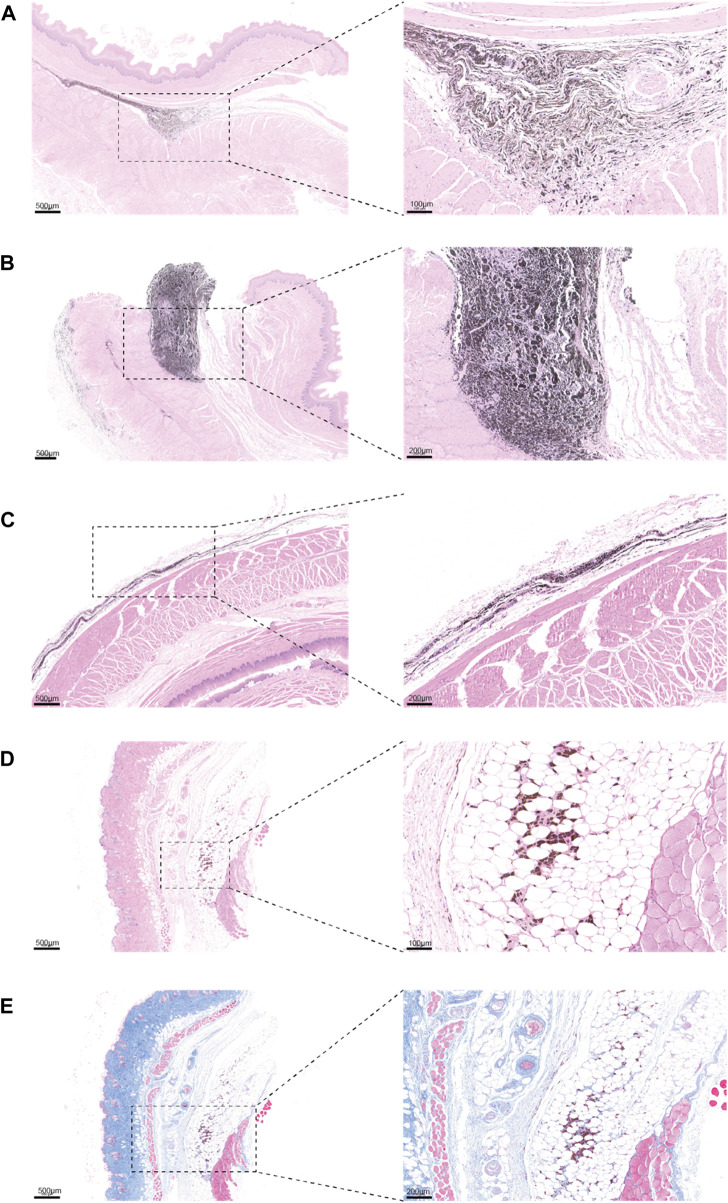
**(A and B)** H&E staining results of the esophagus of a pig after PPy/PVP endoscopic tattooing for 24 days. **(C)** H&E staining results of the rectum of pig after India Ink endoscopic tattooing for 24 days. **(D)** H&E image of KM mice after skin tissue tattooing for 180 days. **(E)** Masson image of KM mice after skin tissue tattooing for 180 days.

Additionally, by examining H&E sections of the tattoo sites on day 24 of the large animal tattoo experiments, we noted that the stability of PPy/PVP as a tattoo dye remained largely unchanged throughout the study period. (Figures 6A,B). In contrast to India Ink tattooing, PPy/PVP exhibited deeper penetration and stability even after 24 days (Figures 6A–C). Concurrently, to evaluate the longevity of tattooing retention and the safety profile of PPy/PVP, comprehensive experiments were conducted using KM mice. This entailed the subcutaneous administration of 100 μL of a PPy tattooing solution with a concentration of 1,000 ppm on day 0. Subsequently, on day 180, the mice were euthanized, and skin tissues at the injection site were meticulously examined, undergoing both H&E staining and Masson staining. The results unveiled the tattooing persistence of PPy/PVP within the dorsal skin tissues even after 180 days, affirming its enduring stability (Figures 6D,E). Furthermore, both H&E and Masson stainings corroborated the favorable safety profile of PPy/PVP, underscoring its suitability for long-term applications with minimal adverse effects. These results proved the PPy/PVP’s potential as a good candidate for endoscopic tattooing and tattooing applications. In summary, our comprehensive findings collectively affirm that PPy/PVP demonstrates commendable biological safety as an endoscopic tattooing dye, which holds great promise for the mini-invasive endoscopic tattooing of the digestive tract.

## Conclusion

As the prevalence of GI lesions continues to escalate, there is a growing demand for endoscopic markers within the GI tract. Tattooing the colon near tumor or polypectomy sites has emerged as a pivotal method for guiding surgical resection or post-polypectomy monitoring. However, conventional dyes used for endoscopic tattooing, such as India ink and methylene blue, present notable drawbacks, including frequent adverse reactions, limited tattooing durations, and high overall costs attributed to the need for subsequent re-tattooing. In this study, we introduced a novel endoscopic tattoo dye, PPy/PVP. Unlike India ink, PPy/PVP is devoid of mugwort, phenol, polycyclic hydrocarbons, ammonia, and gelatin. Moreover, it eliminates the laborious pre-injection treatment required for Indian inks and is manufactured without unpleasant odors. *In vitro* testing has showcased the exceptional stability of PPy/PVP across a broad spectrum of solutions, while comprehensive *in vivo* assessments have validated its superior biosafety. Endoscopic tattooing with PPy/PVP in porcine and kM mouse models yielded no adverse effects, with tattooing effects enduring for at least 24 and 180 days, respectively. PPy/PVP has established its safety and efficacy as a tattooing dye, serving as a crucial tool for endoscopic monitoring of patients who have not undergone surgery. Furthermore, PPy/PVP serves akin to traditional tattooing solutions and is also indicated for colonoscopy, particularly in scenarios where surgical resection is anticipated. Additional research has indicated that PVP not only influences the photothermal properties of the material but also enhances its drug loading capacity. In the near future, PPy/PVP may serve not only as a tattoo pigment but also as a carrier for various drugs, offering improved outcomes and multifunctionality. In summary, PPy/PVP, as a newly developed tattooing dye, offers the benefits of safety, prolonged tattoo duration, and cost-effectiveness, promising diverse applications in the future.

## Data Availability

The original contributions presented in the study are included in the article/supplementary material, further inquiries can be directed to the corresponding authors.

## Abbreviations

PVP, polyvinylpyrrolidone; PPy, polypyrrole; PPy/PVP, polypyrrole/polyvinylpyrrolidone; GI, gastrointestinal; CCK-8, Cell Counting Kit-8; SEM, Scanning electron microscopy; DMEM, Dulbecco’s Modified Eagle Medium; FBS, Fetal Bovine Serum; PBS, Phosphate Buffer Saline; OD, optical density; KM mice, Kunming mice; H&E staining, Hematoxylin and Eosin staining; XRD, X-Ray diffraction; FTIR, Fourier Transform Infrared; DI water, Deionized water; NPs, nanoparticles.
